# Expressing MicroRNA *Bantam* Sponge Drastically Improves the Insecticidal Activity of Baculovirus via Increasing the Level of Ecdysteroid Hormone in *Spodoptera exigua* Larvae

**DOI:** 10.3389/fmicb.2018.01824

**Published:** 2018-08-07

**Authors:** Zihan Ran, Xiaojie Shi, Fangting Han, Jianbei Li, Youyi Zhang, Yanjun Zhou, Juan Yin, Rui Li, Jiang Zhong

**Affiliations:** State Key Laboratory of Genetic Engineering, Department of Microbiology and Microbial Engineering, School of Life Sciences, Fudan University, Shanghai, China

**Keywords:** microRNA, microRNA sponge, microRNA *bantam*, ecdysteroid hormone, baculovirus, bio-pesticide

## Abstract

*Bantam* is a conserved miRNA highly expressed in insects. We previously showed that the antisense inhibitor (antagomiR) of *bantam* improved the infection by baculovirus Autographa californica nucleopolyhedrovirus (AcMNPV) in *Spodoptera exigua* and *S. litura* larvae. Here, we constructed a recombinant AcMNPV (vPH-banS) expressing *bantam* sponge, an mRNA containing eight antisense binding sites for *bantam*. Infection with wild type AcMNPV (WT) or the control recombinant virus vPH resulted in a significant increase of *bantam* level, whereas infection with vPH-banS led to an approximately 40% reduction of *bantam* in both Sf9 cells and *S. exigua* larvae. Although, comparable production of budded virus and polyhedra were detected in vPH-banS-, vPH-, and WT-infected Sf9 cells, vPH-banS showed remarkably increased insecticidal activity in *S. exigua* larvae. The 50% lethal concentration and the median lethal time of vPH-banS was only 1/40 and 1/2, respectively, of both vPH and WT. Further analysis showed that the level of molting hormone 20-hydroxyecdysone (20E) was significantly higher in larvae infected with vPH-banS than those infected with vPH or WT. This was confirmed by the result that the larvae treated with *bantam* inhibitor also had a markedly increased 20E level. Moreover, feeding larvae with 20E increased the virus-mediated mortality, whereas feeding with juvenile hormone partially reverted the high insecticidal effect of vPH-banS. Together, our results revealed that vPH-banS infection suppresses the level of *bantam*, and in turn elevates level of 20E in infected insects, resulting in increased susceptibility to baculovirus infection. Our study provided a novel approach to improve a baculovirus bio-insecticide by interfering with a key homeostasis-regulating miRNA of the host.

## Introduction

Baculoviruses are a group of DNA viruses that infect insects and some other invertebrates (Rohrmann, 2013). They are widely used as vectors for the production of foreign proteins in insect cells, as well as biological insecticides against pest insects. Like many other viruses, successful infection of baculovirus involves delicate interactions with the host. For example, members of baculoviruses encode the enzyme ecdysteroid UDP-glucosyltransferase (EGT) that can inactivate host molting hormone 20-hydroxyecdysone (20E) (O’Reilly and Miller, 1989) and interfere with insect development, resulting in the prolonged survival of infected larvae and the increased production of progeny virus (O’Reilly and Miller, 1991). Viral chitinase and cathepsin are also important for host tissue breakdown and virus dissemination late in the infection (Hawtin et al., 1997).

MicroRNAs (miRNAs) are small non-coding RNAs capable of regulating the expression of multiple target genes post-transcriptionally (Ambros, 2004). They are encoded by most eukaryotes and many viruses, and have important biological functions. In insects, miRNAs have been shown to regulate a variety of physiological processes throughout the life cycle, including molting, metamorphosis, oogenesis, embryogenesis, behavior, and immunity (Asgari, 2013; Lucas et al., 2015).

miRNAs also play important roles in baculovirus infection and virus–host interaction. Virally encoded miRNAs have been found in baculovirus Autographa californica nucleopolyhedrovirus (AcMNPV) (Zhu et al., 2013; Zhu M. et al., 2016), Bombyx mori nucleopolyhedrovirus (Singh et al., 2010, 2012, 2014), and Spodopera litura nucleopolyhedrovirus (Kharbanda et al., 2015), etc., and shown to regulate virus gene expression and replication. Meanwhile, baculovirus infection also alters the expression profiles of host miRNA, suggesting their function in virus–host interactions (Mehrabadi et al., 2013; Shi et al., 2016). For example, Heliocoverpa armigera single nucleopolyhedrovirus infection resulted in the down-regulation of host miR-14, a positive regulator of the nuclear receptor for 20E (Jayachandran et al., 2013), mitigating the effect of 20E.

*Bantam* gene was first identified in the fruit flies *Drosophila melanogaster* and its overexpression caused the overgrowth of wings and eye tissues (Hipfner et al., 2002). Subsequent studies found that it actually encoded a miRNA (Brennecke et al., 2003). In *Drosophila*, *bantam* functions in at least two important processes: preventing apoptosis by down-regulating the apoptotic gene *hid* (Brennecke et al., 2003) and promoting cell proliferation by targeting genes like *mad* (Robins et al., 2005). It has also been reported to facilitate the systemic growth of flies by repressing the release of 20E in the ecdysone-producing cells (Boulan et al., 2013), though the exact mechanism is not known yet. A recent study showed that *bantam* might also target the circadian rhythm gene *clk*, thus regulating 20E level (Lerner et al., 2015).

*Bantam* is also found in lepidopterans, though its role in this group of insects is poorly understood. Mehrabadi et al. (2013) showed that *bantam* was one of the most abundant miRNAs in Sf9 cells. Our previous work found that the level of *bantam* rose significantly both in Sf9 cells and *S. exigua* larvae after AcMNPV infection, and suppressing its level increased the virus infectivity in insects (Shi et al., 2016).

Here, we further examined the role of *bantam* during AcMNPV infection by using a recombinant virus expressing *bantam* sponge, a transcript containing multiple partial binding sites for *bantam* miRNA (Ebert et al., 2007; Kluiver et al., 2012). The recombinant virus showed enhanced insecticidal activity and shorter lethal time than control viruses. Further analysis indicated that the phenomenon was associated with increased 20E level in virus-infected larvae.

## Materials and Methods

### Cells, Viruses, and Insects

The fall armyworm *Spodoptera frugiperda* cell line Sf9 were maintained at 27°C using TNM-FH medium (Sigma-Aldrich, St. Louis, MO, United States) supplemented with 10% FBS and antibiotics (100 U/ml penicillin, and 100 μg/ml streptomycin). The wild-type AcMNPV (strain 1A, WT) and recombinant viruses vPH and vPH-banS (see below) were used in the study. The virus titer was determined by end-point dilution method in Sf9 cells. *S. exigua* (beet armyworm) larvae were reared individually in polymer cups on artificial diet (Keyun Biocontrol, Jiyuan, China), under the condition of 28°C, 70–90% humidity, and the photoperiod of 15L:9D.

### Generation of Recombinant Baculoviruses

**FIGURE 1:**
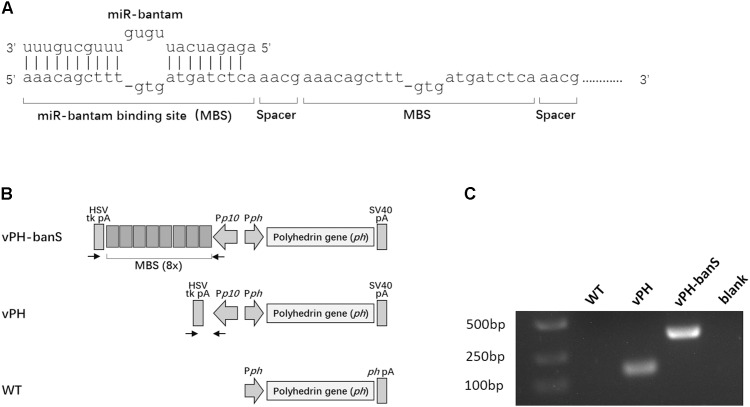
Construction of a recombinant baculovirus expressing miR-*bantam* sponge. **(A)** Design of *bantam* sponge. The sponge contained eight *bantam* antisense binding sites (MBSes). Each MBS contains three mismatches and one deletion. A four-nucleotide spacer (5′-aacg-3′) was inserted between each two MBSes. **(B)** Schematic overview of the *polyherin* gene locus of recombinant viruses vPH-banS, vPH, and wild type AcMNPV (WT). In vPH-banS, the *bantam*-sponge sequence and *polyhedrin* gene were inserted downstream of the *p10* promoter (P*p10*) and the *polyhedrin* gene promoter (P*ph*), respectively. The control virus (vPH) had only the *polyhedrin* gene. Arrows indicate the locations of primers used for PCR verification of the virus constructs. **(C)** PCR verification of virus constructs. DNA extracted from virus-infected Sf9 cells was used as PCR template.

Recombinant baculoviruses were constructed using Bac-to-Bac system (Life Technology, Carlsbad, CA, United States). The coding sequence of AcMNPV *polyhedrin* gene was amplified from wild type AcMNPV DNA using the primers 5′-ttgaattctattttactgaattcgtaacagttttgt-3′ and 5′-tttctagagcacagaatctagagcttaataaatgta-3′ (*Eco*RI and *Xba*I sites are underlined) and cloned into *EcoR*I and *Xba*I-doubly digested pFsatbac-Dual (Life Technology, Carlsbad, CA, United States), yielding pFBdual-PH. A *bantam*-sponge sequence, which contained eight repeats of *bantam* binding sequence (**Figure 1A**) and restriction enzyme sites of *Nhe*I and *Xho*I at either end of the fragment, was chemically synthesized (Sangon Biotech, Shanghai, China) and inserted into the two restriction sites downstream of the *p10* promoter in pFBdual-PH, yielding pFBdual-PH-banS. Both pFBdual-PH and pFBdual-PH-banS were used to generate recombinant baculoviruses vPH and vPH-banS, respectively, using Bac-to-Bac^TM^ Baculovirus Expression System. The schematic diagram of the *polyhedrin* gene locus in these viruses is shown in **Figure 1B**. Recombinant viruses were confirmed by PCR using viral DNA extracted from the infected culture medium as the template and the primers 5′-gtgtttcagttagcctccc-3′ and 5′-gtgtttcagttagcctccc-3′.

### RT-qPCR Quantification of *Bantam* Expression

To determine the level of *bantam* in Sf9 cells, total small RNAs (<200 nt) were harvested from Sf9 cells and reverse-transcribed using miRVana microRNA Isolation Kit (Ambion, Life Technologies, Carlsbad, CA, Unites States). RT-qPCR detection of bantam was performed using miScript II RT Kit (Qiagen, Venlo, Netherlands) according to the manufacturer’s instructions. The forward primer of *bantam* and the internal control (U6 snRNA) were 5′-ctttctgagatcattgtgaaag-3′ and 5′-agagacgattagcatggccc-3′, respectively. The reaction was carried out on Stratagene MX3000p and data processing was done with the software MxPro. Relative expression of miRNA was normalized to *U6* RNA by 2^-ΔΔt^ method. Similar procedures were used to determine *bantam* expression in *S. exigua* insects, except that the larvae were first ground in liquid nitrogen.

### Determination of Viral DNA in Polyhedra and Larvae

Polyhedra were obtained from infected larvae and washed three times with PBS. The same amount of polyhedra of vPH-banS, vPH, and WT was left in alkaline solution (10 mM Na_2_CO_3_-50 mM NaCl) for 2 h at room temperature. The suspension was then centrifuged at 2,600 × *g* for 15 min to remove undissolved debris. The supernatant (500 μl) was mixed with equal volume of lysate buffer (10 mM Tris–HCl, 10 mM EDTA, 0.25% SDS), and 5 μl proteinase K (20 mg/ml), and placed at 50°C for 2 h. Viral DNA was extracted with phenol-chloroform and precipitated with ethanol. The amount of viral DNA was determined by qPCR using the primers targeting viral *gp41* gene: 5′-agagttgggacagagcaacg-3′, 5′-gcgccaccgttgtaaaactt-3′. To determine the virus replication in insects, the infected larvae were ground in liquid nitrogen, and total DNA were extracted using QiaAMP DNA Mini Kit (Qiagen). The level of viral DNA was determined by qPCR as above, and normalized against host *tubulin* gene.

### Insect Experiments

Viruses were propagated in *S. exigua* larvae, and the polyhedra isolated from infected larvae were used in experiments. To determine the 50% lethal concentration (LC_50_) of each virus, polyhedra suspensions of different concentrations were sprayed on the surface of small pieces of diet to feed newly molted third instar larvae. New diet was added 24 h later when the contaminated diet was consumed completely. Mortality and pupation were recorded daily. *Bantam* antagomiR, a chemically modified antisense oligonucleotide of *bantam*, was designed, synthesized, and used to suppress the level of *bantam* in larvae as described previously (Shi et al., 2016). To interfere with the internal hormones in insect, 20E or juvenile hormone (JH, Lvfeng Agriculture and Sericulture Base, China) were dissolved in sterile water, and the solution was evenly sprayed on the diet daily.

### Determination of 20E Level in Insect

Larvae were weighted and phosphate buffered saline (PBS) was added to each larva according to the weight (90 μl per 10 mg of larval weight). They were then homogenized individually and centrifuged at 2 600 × *g*, 4°C, for 15 min. The supernatant was collected and 20E level was determined using Insect 20-Hydroxyecdysone ELISA Kit (MLBIO Biotechnology, Shanghai, China).

### Fluorescence *in situ* Hybridization (FISH)

FISH experiment was carried out following a standard protocol (Pena et al., 2009). Briefly, the fat body was dissected from insects and fixed in 4% paraformaldehyde (PFA) (Sigma-Aldrich, St. Louis, MO, United States). It was then permeabilized with proteinase K (20 μg/ml in PBS prepared with RNase free water) for 15 min, and post-fixed in 4% PFA for 10 min, followed by treatment with prehybridization solution. For hybridization, 4 pmol of Cy3-labeled probe (*bantam*: 5′tagctttcacaatgatctca3′, mock: 5′cagtacttttgtgtagtacaa3′, synthesized and modified by GenePharma, Shanghai, China) in 100 μl hybridization buffer (prehybridization solution with 10% dextran sulfate) were applied per section, and incubated in a sealed humidified chamber for 16 h. Both prehybridization and hybridization were carried out at 27°C, 20°C below the Tm. After stringency washes, the slides were stained briefly with 6-diamidino-2-phenylindole (DAPI) for 5 min in dark. The samples were visualized using a Leica TCS SP8 epifluorescence microscope and LAS AF software (Leica Microsystems, Gateshead, United Kingdom).

### Scanning Electron Microscope

Polyhedra were harvested from the larvae infected with vPH, vPH-banS, and WT, and washed three times with PBS. The polyhedra suspension was dropped on conductive tapes, and left at room temperature for 15 min. Then it was coated with a thin layer of gold and examined with a Hitachi TM3000 scanning electronic microscope.

### Statistical Analysis

Unless otherwise stated, bars represent means ± SD, and averages were compared using a bidirectional unpaired Student’s *t*-test. The showed results are representative of at least three independent experiments.

## Results

### Construction of Recombinant Baculovirus Containing *Bantam* Sponge

Our previous work showed that *bantam* inhibitor increased the insect mortality caused by baculovirus AcMNPV in both *S. exigua* and *S. litura* larvae (Shi et al., 2016). To further understand the role of *bantam* in virus replication and virus–host interaction, a *bantam*-specific sponge sequence was designed and inserted into the AcMNPV genome. The sponge sequence contained eight partial antisense binding sites for *bantam*, each with one deletion and three mismatches. A four-nucleotide spacer (5′-aacg-3′) was inserted between every two binding sites (**Figure 1A**). The sponge sequence was chemically synthesized and inserted downstream of the *p10* promoter of the transfer vector pFastBac-Dual. The *polyhendrin* gene was inserted downstream of the *polyhedrin* promoter in the same vector. The recombinant baculovirus, vPH-banS (**Figure 1B**), was obtained using the Bac-to-Bac system. A control recombinant virus with only *polyhedrin* gene downstream of the *polyhedrin* promoter (vPH) was also constructed (**Figure 1B**). PCR and sequencing results confirmed the successful construction of vPH-banS and vPH (**Figure 1C**).

### vPH-banS Suppressed *Bantam* Level in Sf9 Cells

**FIGURE 2:**
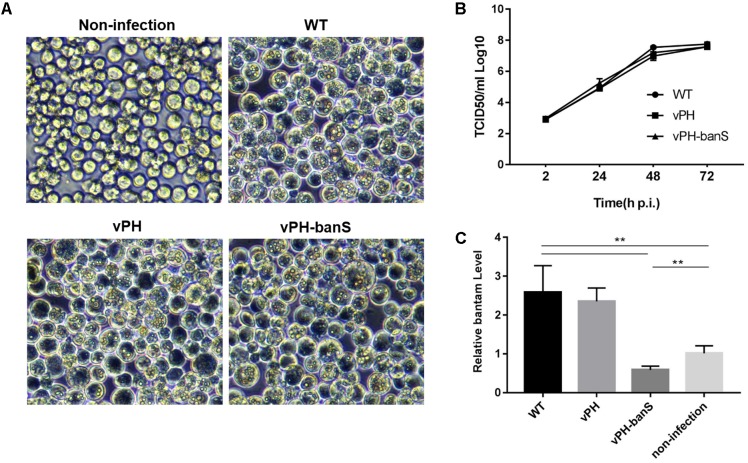
vPH-banS showed similar replication kinetics as the control viruses but suppressed *bantam* level in Sf9 cells. **(A)** Microscopic observation of virus-infected cells. Sf9 cells were infected with vPH-banS, vPH, and WT (multiplicity of infection, MOI = 0.5) and examined 96 h p.i. Representative views are shown. **(B)** The kinetics of BV production. The titers of BV in culture medium at different times post infection were determined by end-point dilution. **(C)** The level of miRNA *bantam* in virus-infected cells. Total small RNA samples were prepared from virus infected or non-infected cells 72 h p.i. The levels of *bantam* were determined with real-time RT-PCR using *U6* RNA as the internal control. Fold change compared to the non-infected control is shown. (^∗∗^*p* < 0.01).

vPH-banS, vPH, and WT were first compared for their infectivity in Sf9 cells. Similar level of polyhedra formation and budded virus (BV) production were observed for all three viruses (**Figures 2A,B**), though cells infected with vPH-banS had slightly higher level of *polyhedrin* mRNA and protein (data not shown). These results were consistent with our previous study using *bantam* inhibitor (Shi et al., 2016).

We further examined *bantam* expression in Sf9 cells by qPCR. It was found that *bantam* level increased by about 2.5-fold after WT and vPH infection, which was also consistent with our previous study (Shi et al., 2016). However, in vPH-banS-infected cells, the *bantam* level was only about 60% of that in uninfected cells (**Figure 2C**), indicating that *bantam* sponge effectively suppressed *bantam* level in Sf9 cells.

### vPH-banS Suppressed *Bantam* Level in *S. exigua* Larvae

**FIGURE 3:**
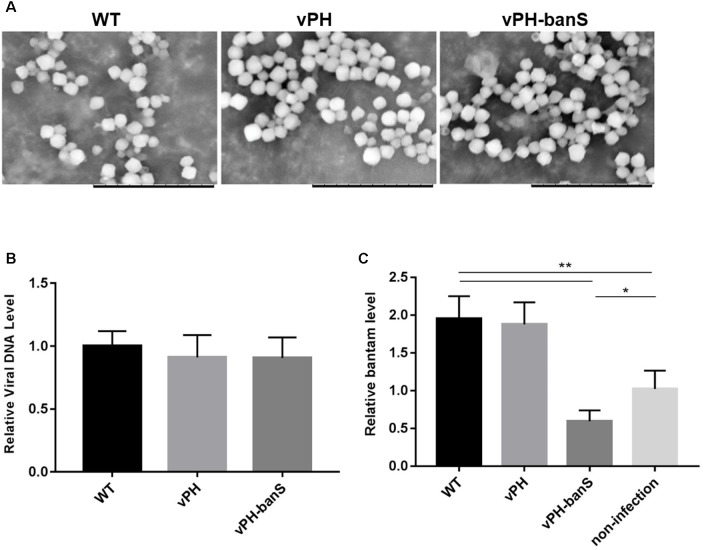
vPH-banS suppressed the level of *bantam* in *S. exigua* larvae. **(A)** Morphological comparison of vPH-banS, vPH, and WT polyhedra produced in larvae by scanning electron microscopy. The scale bar represents 10 μm. **(B)** Viral DNA content in virus polyhedra. Total DNA was extracted from the same amount of vPH-banS, vPH and WT polyhedra, and compared quantitatively by qPCR using a pair of primers targeting the viral *gp41* gene. **(C)** *bantam* level in infected larvae. Total RNA was extracted from whole body of larvae infected with vPH-banS, vPH, and WT (1 × 10^6^ polyhedra/ml), 4 d p.i., and *bantam* level was determined by real-time RT-PCR using *U6* RNA as the internal control. Fold change compared to the non-infected control is shown (^∗^*p* < 0.05; ^∗∗^*p* < 0.01).

The polyhedra of vPH-banS, vPH, and WT viruses were isolated from infected *S. exigua* larvae and visualized using scanning electron microscope. As shown in **Figure 3A**, the polyhedra generated by the three viruses were similar in size and morphology. They also contained similar amount of viral DNA, as determined by qPCR (**Figure 3B**), indicating that *bantam* sponge did not affect polyhedra morphogenesis.

Third instar *S. exigua* larvae were then inoculated with the same concentration of polyhedra (1 × 10^6^polyhedra/ml) of vPH-banS, vPH, and WT, and the level of *bantam* was compared 4 d p.i. Similar to the results in Sf9 cells, infection with WT and vPH resulted in significant increase in *bantam* level, whereas infection with vPH-banS suppressed *bantam* level by about 40% compared to the mock-infected larvae (**Figure 3C**).

### vPH-banS Had Increased Insecticidal Activity in *S. exigua*

**FIGURE 4:**
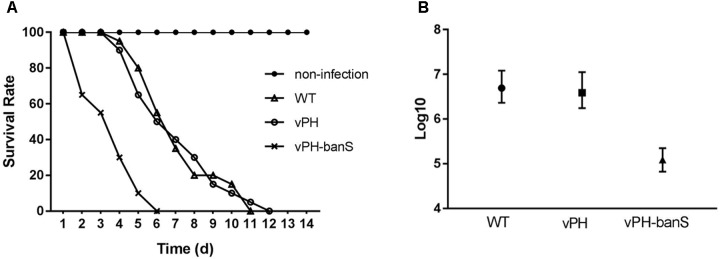
vPH-banS had increased insecticidal activity in *S. exigua* larvae. **(A)** Second instar larvae of *S. exigua* were infected with viruses (2 × 10^7^ polyhedra/ml) and the mortality was recorded daily. Each group included 30 or more larvae. **(B)** Early third instar larvae were inoculated with five different concentrations of polyhedra of vPH-banS, vPH, and WT. The infection concentrations were: 4.0 × 10^7^, 8.0 × 10^6^, 1.6 × 10^6^, 3.2 × 10^5^, 6.4 × 10^4^ polyhedra/ml for WT, 2.8 × 10^7^, 5.5 × 10^6^, 1.1 × 10^6^, 2.2 × 10^5^, 4.4 × 10^4^ polyhedra/ml for vPH, and 4.6 × 10^6^, 9.2 × 10^5^, 1.6 × 10^5^, 3.7 × 10^4^, 7.4 × 10^3^ polyhedra/ml for vPH-banS, respectively. Each group included 20 or more larvae. Mortality was recorded. LC_50_ with lower and upper 95% fidelity is shown.

The insecticidal activity of vPH-banS was further examined by infecting late second instar *S. exigua* larvae with polyhedra at 2 × 10^7^ polyhedra/ml. As shown in **Figure 4A**, vPH-banS killed larvae much faster than WT and vPH, with the median lethal time decreased from 7.0 (WT) and 7.2 (vPH) days to 3.6 days (**Figure 4A**). vPH-banS also had markedly reduced LC_50_, which decreased from 3.9 × 10^6^ (with lower and upper 95% fidelity of 1.7 × 10^6^ and 1.1 × 10^7^, respectively) of vPH, and 4.9 × 10^6^ (2.3 × 10^6^, 1.2 × 10^7^) of WT, to 1.2 × 10^5^ (6.6 × 10^4^, 2.2 × 10^5^) polyhedra/ml of vPH-banS (**Figure 4B**).

**FIGURE 5:**
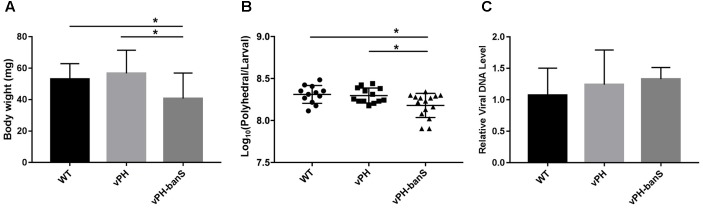
vPH-banS inhibited larval growth and polyhedra production. The early third instar larvae of *S. exigua* were infected with WT, vPH, and vPH-banS (1.0 × 10^6^ polyhedra/ml). **(A)** Larval weights were recorded 5 d p.i. **(B)** Polyhedra productions were counted after larval death. **(C)** Viral DNA replication was determined by qPCR using primers targeting virus *gp41* gene. Host *tubulin* gene was used as the internal control. Each group had 15 larvae for **(A,B)**, and 8 larvae for **(C)** (^∗^*p* < 0.05).

The replication of the three recombinant viruses in *S. exigua* larvae was further compared. Infection with vPH-banS showed lower body weight 5 d p.i., and lower polyhedra production than that in vPH and WT (**Figures 5A,B**), whereas all three viruses had similar levels of viral DNA (normalized against host *tubulin* gene) 5 d p.i. (**Figure 5C**). These results suggested that the high infectivity of vPH-banS is mediated by its effect on host growth, rather than a change in virus replication.

### vPH-banS Infection Resulted in Increased 20E Level

**FIGURE 6:**
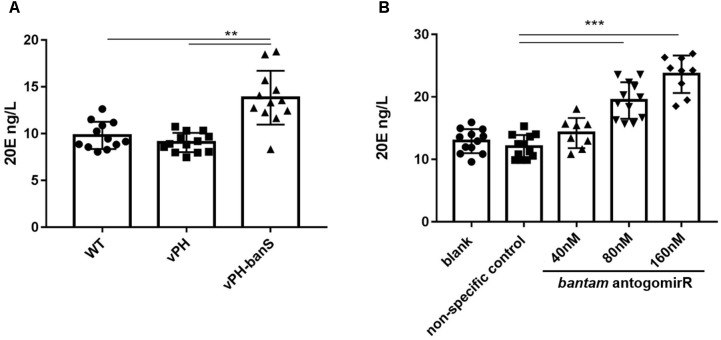
Inhibition of *bantam* increased 20E level in *S. exigua* larvae. **(A)** Newly molted third instar larvae were infected with WT, vPH, and vPH-banS (2 × 10^5^ polyhedra/ml) and the level of 20E was measured by ELISA 5 d p.i. **(B)** second instar larvae were treated with 40, 80, or 160 nM *bantam*-antagomiR, and the level of 20E was determined at the end of third instar. Each group included 20 or more larvae. (^∗∗^*p* < 0.01; ^∗∗∗^*p* < 0.001).

In *Drosophila*, *bantam* was reported to repress the release of ecdysteroid hormone (Boulan et al., 2013). To examine if the suppression of *bantam* by vPH-banS would affect 20E level in *S. exigua*, newly molted third instar larvae were infected with sub-lethal dose of vPH-banS, vPH, and WT (2 × 10^5^ polyhedra/ml) and 20E level was measured 5 d p.i. As can be seen in **Figure 5A**, vPH-banS-infected insects had significantly higher level of 20E than those infected with WT or vPH (**Figure 6A**).

Further, second instar larvae were administrated with different concentrations of *bantam* antagomiR, a chemically modified antisense inhibitor of bantam miRNA (Shi et al., 2016), and 20E level was measured at the end of third instar. The results showed that the production of 20E increased with the concentration of *bantam* antagomiR in a dose-dependent manner (**Figure 6B**). These data suggested that the increase of 20E after vPH-banS infection is related to the suppression of *bantam*.

### The Levels of *Bantam* and 20E Were Reversely Correlated in *S. exigua* Development

**FIGURE 7:**
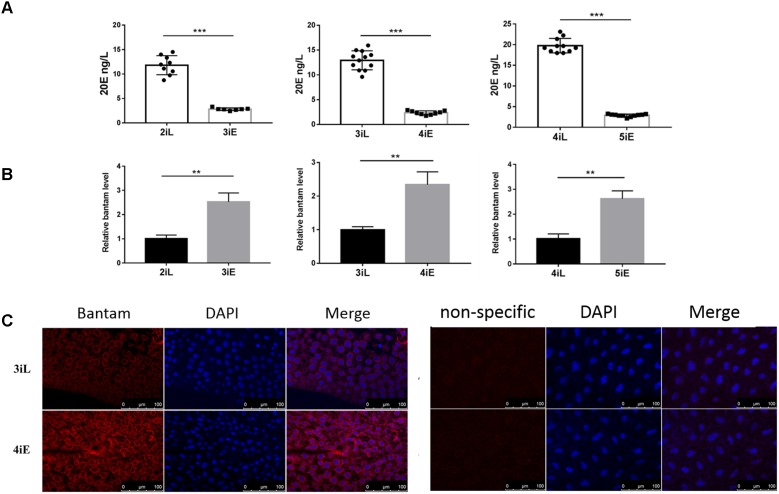
The levels of *bantam* and 20E were reversely correlated with each other in *S. exigua*. **(A,B)** The levels of 20E **(A)** and *bantam* **(B)** in *S. exigua* larvae at different developmental stages were measured by qPCR and ELISA. 2iL, 3iL, 4iL: late second, third, or fourth instar, respectively. 3iE, 4iE, 4iE: newly molted early third, fourth, or fifth instar, respectively. **(C)** *Bantam* expression in *S. exigua* larvae was detected by Cy3-labeled DNA probe through *in situ* hybridization of late third or early fourth instar larvae. (^∗∗^*p* < 0.01; ^∗∗∗^*p* < 0.001).

Next, the levels of *bantam* and 20E were measured in late second, third and fourth instar larvae before molting, and in newly molted early third, fourth, and fifth instar larvae. We found that *bantam* expression was high at the beginning of each instar, and low at the end of each instar, whereas the level of 20E followed an opposite pattern, which was low at the beginning of each instar, and high at the end of each instar (**Figures 7A,B**). Much stronger fluorescence was also observed in the tissues of early fourth instar larvae than that of late third instar larvae when examined by fluorescent *in situ* hybridization using Cy3-labeled probe against *bantam* (**Figure 7C**). These observations provided further evidence that *bantam* may also regulate 20E level in *S. exigua*.

### JH Partially Eliminated *Bantam* Sponge-Mediated Increase of Insecticidal Activity of vPH-banS

**FIGURE 8:**
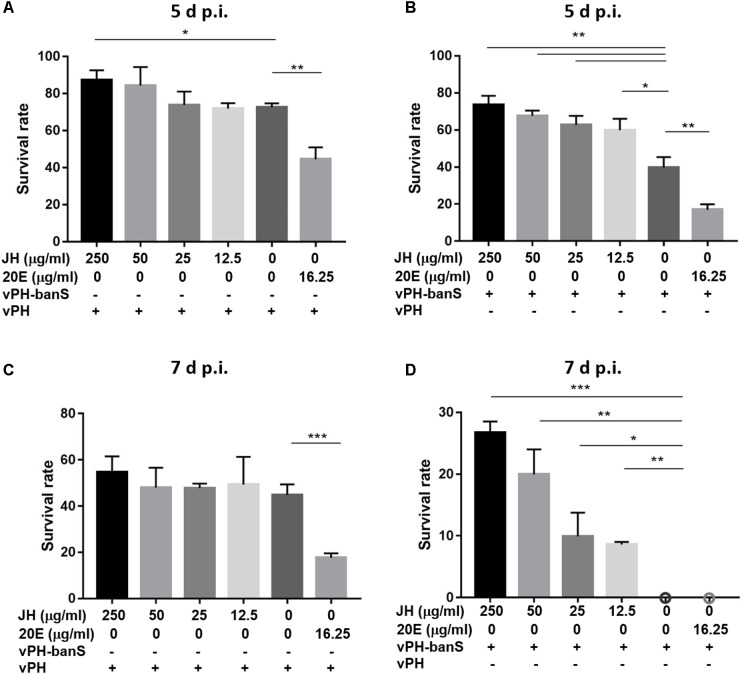
The effect of juvenile hormone (JH) on the insecticidal activity of vPH-banS and vPH. Early third instar larvae of *S. exigua* were infected with vPH **(A,C)** and vPH-banS **(B,D)** (1.0 × 10^6^ polyhedra/ml), supplemented with 20E (16.25 μg/ml) or different concentrations of JH (250, 50, 25, or 12.5 μg/ml). Survival rates were recorded 5 d p.i. **(A,B)** and 7 d p.i. **(C,D)**. 30 or more larvae per group were assayed. (^∗^*p* < 0.05; ^∗∗^*p* < 0.01; ^∗∗∗^*p* < 0.001).

To further characterize the mechanisms underlying *bantam*-mediated insecticidal activity, early third instar larvae were infected with vPH-banS and vPH (1 × 10^6^ polyhedra/ml), together with 20E (16.25 μg/ml), or different concentrations of JH (12.5, 25, 50, 250 μg/ml) in the diet. As shown in **Figure 8**, 20E supplementation further reduced insect survival to a similar degree in vPH-banS and vPH. Meanwhile, the addition of JH only slightly increased larval survival in vPH-infected larvae, whereas markedly increased larval survival in vPH-banS group. At 5 d p.i., the survival rate of vPH-banS-infected larvae increased from 39.8 to 73.6% with 250 μg/ml JH supplementation. At 7 d p.i., all of the larvae succumbed to vPH-banS infection, however, 250 μg/ml JH supplementation led to 26.8% survival in vPH-banS infected larvae. Since 20E and JH are thought to counteract with each other in regulating the growth and death of insect cells, the data support the notion that the elevated level of 20E accounted for the increased infectivity of vPH-banS.

## Discussion

*Bantam* is a miRNA found specifically in insects and other invertebrates. Apart from *Drosophila*, *bantam* has also been reported in blood fluke *Schistosoma japonica* (Xue et al., 2008; Zhu L. et al., 2016) and silkworm *B. mori* (Liu et al., 2010). About 700 genes potentially regulated by *bantam* have been reported in *Drosophila* (Delanoue and Léopold, 2013), among which five targets have been properly identified: *hid* (Brennecke et al., 2003), *mei-P26* (Herranz et al., 2010), *enabled* (Becam et al., 2011), *capicua* (Herranz et al., 2012a), and *socs36E* (Herranz et al., 2012b). Multiple functions have been attributed to *bantam*, including cell cycle regulation, body development, apoptosis, etc. Moreover, Boulan et al. found that *bantam* represses ecdysone release in ecdysone-producing cells in *Drosophila* (Boulan et al., 2013). Our previous work suggested that *bantam* is potentially involved in baculovirus infection of lepidopteran insects (Shi et al., 2016).

In the current work, we constructed a recombinant baculovirus expressing a *bantam* sponge, vPH-banS, and the control virus vPH. Both viruses had *polyhedrin* genes and formed polyhedra during infection so that they could infect insects *per os*. miRNA-sponge has been reported to be efficient in reducing the effective level of specific miRNAs in cells (Rybak et al., 2008; Sayed et al., 2008; Horie et al., 2009), in some cases even to an extent that was undetectable by northern blot (Rybak et al., 2008). Sponges with imperfect miRNA binding sites, i.e., binding sites that include a four-nucleotide central bulge (“bulged sponges”), are reported to be more effective than sponges with perfect match, since such imperfect binding will not lead to the degradation of the sponge RNA (Ebert et al., 2007; Gentner et al., 2008; Kumar et al., 2008).

The *bantam* sponge was effective in reducing *bantam* level both *in vivo* and *in vitro*. AcMNPV infection resulted in the increase of *bantam* level, whereas the infection of vPH-banS significantly reduced *bantam* level in Sf9 cells and *S. exigua* larvae. This remarkable effect might be due to the removal of the sponge-sequestered *bantam* miRNA during miRNA isolation, or due to sponge-triggered *bantam* degradation. Nevertheless, the low *bantam* level had no major effect on virus replication in Sf9 cell, in terms of both BV production and polyhedra formation, in consistence with our previous study (Shi et al., 2016).

However, vPH-banS showed a drastically increased insecticidal activity in *S. exigua* larvae. The median lethal time and the medium lethal concentration of vPH-banS was only half and one fortieth of the controls, respectively. These were also consistent with our previous study using *bantam* inhibitor (Shi et al., 2016), and suggested that *bantam* is associated with AcMNPV infectivity or host susceptibility. Larvae infected with vPH-banS had lower body weight and less polyhedra production than those infected with control viruses, which could be the result of accelerated infection process. Virus replication, when normalized against host *tubulin* gene, was not affected.

Apart from baculovirus *p10* promoter, we also used insect *U6* promoter and baculovirus *ie2* promoter to drive the expression of *bantam* sponge. Although, increased insecticidal activity was seen for all three viruses, the virus with *p10* promoter, vPH-banS, performed best (data not shown). It seems that the high expression level is important for *bantam* sponge to take effect.

Insect development is coordinately regulated by insect hormones, among which 20E and JH are of particular importance (Dubrovsky, 2005). As the molting hormone, 20E accumulates in the late phase of each larval instar and is responsible for the initiation of molting and metamorphosis. It binds to EcR and turns on the expression of a series of 20E-responsive genes involved in histolysis and tissue regeneration (Dubrovsky, 2005). JH plays important roles in maintaining larval characteristics and preventing metamorphosis. It remains present during the complete larval stage until the last molting and its absence is critical for the transformation both from larval to pupal and from pupal to adult stage (Jindra et al., 2013). 20E and JH are thought to counteract with each other in regulating insect cell growth and death (Liu et al., 2009; Cai et al., 2014), as well as in immunity against infection (Regan et al., 2013; Schwenke et al., 2016).

Since *bantam* has been suggested to regulate host ecdysone hormone release in *Drosophila* (Boulan et al., 2013), we further examined the level of 20E during the infection. Significantly higher levels of 20E was found in vPH-banS-infected larvae than vPH- and WT-infected larvae. Treating insects with *bantam* antagomiR also markedly increased the levels of 20E. Thus, our data suggests that, as in *Drosophila*, *bantam* negatively regulates 20E level in *S. exigua*, though the exact mechanism is not clear and warrants further investigation.

It has long been known that 20E has a role in baculovirus infection. Many members of baculoviruses encode the gene *egt*, which can interact with 20E in the host. Expression of *egt* suppresses the level of 20E and extends the host survival so as to increase the production of progeny virus (Flipsen et al., 1995; O’Reilly, 1995). The increase in *bantam* level after AcMNPV infection seems to have the same effect as *egt*, which could be another example of virus manipulating host physiology in favor of its own replication and dissemination.

The high level of 20E after vPH-banS infection is likely to be the most important factor for its high insecticidal activity. This was supported by the results that feeding larvae with 20E significantly increased virus-mediated larval mortality, whereas feeding with JH partially reduced the high mortality in vPH-banS group. Taken together, our data further demonstrated *bantam* as an important host defending factor in response to baculovirus infection, and suppression of *bantam* by its sponge led to enhanced 20E level, thus contributing to the increased susceptibility to virus infection.

Several mechanisms may account for the enhancement of virus infection by 20E. One possible reason is that 20E acts directly on virus infection. Zhou et al. (2002) reported that the promoter of BmMNPV immediate early gene *ie1* had increased transcriptional activity in the presence of 20E, though its effect on virus replication was not studied *in vitro* or *in vivo*. Alternatively, 20E might function through inhibiting cellular activity and inducing cell apoptosis. As the molting hormone, 20E binds to the nuclear receptor EcR, represses Myc level to inhibit the general insulin/IGF signaling, and limits cell proliferation and tissue growth (Colombani et al., 2005; Mirth et al., 2005). 20E has also been reported to induce cell apoptosis, and this activity could be abolished by JH (Liu et al., 2009; Cai et al., 2014). In addition to 20E, inhibition of *bantam* might induce apoptosis by relieving *bantam*-mediated suppression of *hid* expression (Brennecke et al., 2003). Furthermore, 20E might enhance virus infection by inhibiting host immune capability against infection. Studies have shown that JH and 20E had opposite effects on immunity in many insects. 20E inhibited host immunity against infection, whereas JH might counteract this effect (Regan et al., 2013; Schwenke et al., 2016). However, the role of JH and 20E on host immunity against baculovirus infection has not been reported so far and could be an interesting area for future research.

In summary, we demonstrated that miRNA *bantam* modulates virus–host interaction through regulating the level of the molting hormone. Suppressing *bantam* led to the increase of 20E and resulted in increased mortality in virus-infected *S. exigua*. Baculoviruses are considered as bio-pesticides for agricultural pests. However, their slow mode of action has limited their utilization. Efforts have been made to improve their virulence. With further optimization, the *bantam* sponge-expressing recombinant baculovirus may have a great potential in the applications.

## Author Contributions

JZ and RL contributed conception and design of the study. ZR, XS, FH, JL, YOZ, YAZ, and JY performed the experiments. ZR, RL, and JZ wrote the manuscript. All authors read and approved the submitted version.

## Conflict of Interest Statement

The authors declare that the research was conducted in the absence of any commercial or financial relationships that could be construed as a potential conflict of interest.
